# Does an innovative PT program targeting mobility work for Veterans with executive function deficit?

**DOI:** 10.1093/geroni/igaf122.2290

**Published:** 2025-12-31

**Authors:** Elisa Ogawa, Rebekah Harris, Ildiko Halasz, William Milberg, Jonathan Bean

**Affiliations:** VA Boston Healthcare System, Boston, Massachusetts, United States; VA Boston Healthcare System, Boston, Massachusetts, United States; VA Boston Health Care System, Boston, Massachusetts, United States; VA Boston Health Care System, Boston, Massachusetts, United States; Harvard Medical School, Boston, Massachusetts, United States

## Abstract

People with executive function problems may respond differently to Physical Therapy (PT) treatment compared to those without executive function problems. The study aimed to examine the treatment response after a novel PT treatment among Veterans with and without executive function deficits (EFD+/EFD-). This study was a preplanned secondary analysis of a Randomized Controlled Trial among middle- and older-aged Veterans (≥50yrs) with slow gait speeds. Changes in mobility (Short Physical Performance Battery (SPPB), gait speed) and executive function (Delis-Kaplan Executive Function System Verbal Fluency (letter fluency, category fluency, category switching, category switching accuracy) after 8-week PT treatment were evaluated based on baseline EFD status. One-hundred-and-one Veterans who completed the 8-week PT treatment were included in this study (mean age 71) and 15% (n = 15) were EFD+. There was no evidence of differences in mobility outcomes based on EFD status, both exceeding clinically meaningful improvements (Δ SPPB: EFD+ 1.5±1.4 vs. EFD- 1.1±1.6, p = 0.43; Δ gait speed: EFD+ 0.9±9.14 vs. EFD- 0.10±0.15, p = 0.86). We observed statistically significant differences in the change of category switching (EFD+ 2.7±3.1 vs. EFD- -0.5±3.0, p < 0.001) and category switching accuracy (EFD+ 2.9±3.3 vs. EFD- -0.2±2.9, p < 0.001), favoring EFD+. LLWS treatment may be appropriate for improving mobility in individuals with and without EFD. LLWS treatment may also have benefits in aspects of executive function such as cognitive flexibility, particularly for Veterans with EFD+. Future research is needed to evaluate rehabilitation treatment responses based on cognition on a larger sample, given the high prevalence of cognitive impairment among patients with mobility problems.

